# CCL2/MCP-1 modulation of microglial activation and proliferation

**DOI:** 10.1186/1742-2094-8-77

**Published:** 2011-07-05

**Authors:** Ara E Hinojosa, Borja Garcia-Bueno, Juan C Leza, Jose LM Madrigal

**Affiliations:** 1 Departamento de Farmacología, Facultad de Medicina, Universidad Complutense de Madrid, Av. Complutense s/n, 28040 Madrid, Spain; 2 Centro de Investigación Biomédica en Red de Salud Mental (CIBERSAM), and Instituto de Investigación Sanitaria Hospital 12 de Octubre, Madrid, Spain

## Abstract

**Background:**

Monocyte chemoattractant protein (CCL2/MCP-1) is a chemokine that attracts cells involved in the immune/inflammatory response. As microglia are one of the main cell types sustaining inflammation in brain, we proposed here to analyze the direct effects of MCP-1 on cultured primary microglia.

**Methods:**

Primary microglia and neuronal cultures were obtained from neonatal and embryonic Wistar rats, respectively. Microglia were incubated with different concentrations of recombinant MCP-1 and LPS. Cell proliferation was quantified by measuring incorporation of bromodeoxyuridine (BrdU). Nitrite accumulation was measured using the Griess assay. The expression and synthesis of different proteins was measured by RT-PCR and ELISA. Cell death was quantified by measuring release of LDH into the culture medium.

**Results:**

MCP-1 treatment (50 ng/ml, 24 h) did not induce morphological changes in microglial cultures. Protein and mRNA levels of different cytokines were measured, showing that MCP-1 was not able to induce proinflammatory cytokines (IL-1β, IL6, MIP-1α), either by itself or in combination with LPS. A similar lack of effect was observed when measuring inducible nitric oxide synthase (NOS2) expression or accumulation of nitrites in the culture media as a different indicator of microglial activation. MCP-1 was also unable to alter the expression of different trophic factors that were reduced by LPS treatment. In order to explore the possible release of other products by microglia and their potential neurotoxicity, neurons were co-cultured with microglia: no death of neurons could be detected when treated with MCP-1. However, the presence of MCP-1 induced proliferation of microglia, an effect opposite to that observed with LPS.

**Conclusion:**

These data indicate that, while causing migration and proliferation of microglia, MCP-1 does not appear to directly activate an inflammatory response in this cell type, and therefore, other factors may be necessary to cause the changes that result in the neuronal damage commonly observed in situations where MCP-1 levels are elevated.

## Background

One of the first steps necessary for the development of an inflammatory response is the attraction of certain types of cells to the site of inflammation [1]. Multiple factors are involved in this process; among them, chemokines constitute some of the main agents since they are responsible for the creation of the concentration gradient towards which leukocytes and other cells migrate [2]. In agreement with this, the suppression of chemokines, or of their activity, results in a concomitant suspension of the inflammatory response [3].

An exaggerated inflammatory response can cause damage to cells that are necessary for the correct functioning of the tissue/organ they are part of. This is particularly important in brain, where damage to or loss of constitutive cells can have worse consequences than in different tissues with higher regenerative capacities. The main immune cells in brain are microglia [4]; they continuously inspect their environment and react to changes that could threaten homeostasis [5]. This reaction can be detected mainly by changes in their morphology as well as by their expression of different genes or release of certain cytokines. The principal purpose of this reaction appears to be protection of neurons and their synapses [6,7]. However, neurons in the proximity of activated microglia can be damaged by an exaggerated inflammatory response. Microglia can also receive some signals that make them attack healthy neurons as is the case in neurodegenerative diseases such as Alzheimer's disease, HIV-associated dementia or multiple sclerosis, among others [8].

As a consequence of these facts, it becomes interesting to elucidate the precise mechanisms regulating microglial changes and to distinguish between those that have potentially deleterious consequences and those that result in protection against injuries. As surveillance agents, microglia need to travel to the places where they are needed, and chemokines regulate these movements [9]. As the monocyte chemoattractant protein (CCL2/MCP-1) is one of the main chemokines regulating microglial movement, it was our goal to study here some of the effects MCP-1 has on isolated microglia, and to determine if the responses it provokes in microglia may be responsible for the neuronal damage observed in situations where microglia are attracted to sites of inflammation by this chemokine. Different studies have shown that suppression of MCP-1 or of its effects can be protective against different injuries or diseases [10], and have suggested that this could be considered as a therapeutic strategy.

However, an alternative role of MCP-1 depicts a different scenario: MCP-1 has also been proven to protect neurons against different stimuli [11-13], and recent studies have indicated that it could also have other functions besides those that characterize it as a chemoattractant [14-17].

Our experiments show that MCP-1 induces proliferation of isolated microglia, but we could not detect changes in the production of proinflammatory cytokines in microglia treated with similar concentrations of MCP-1. Accordingly, MCP-1 did not induce morphological changes in microglia nor did it induce expression of the proinflammatory enzyme NOS2 or the accumulation of nitrites, an indicator of the production of nitric oxide caused by NOS2. Lipopolysaccharide (LPS) was used as a positive control to cause those changes characteristic of microglia that lead to the production of neurotoxic factors. While LPS also reduced the expression of various neurotrophic factors, MCP-1 did not modify them. All these data suggest that MCP-1 has some effects on microglia which do not appear to be directly toxic to neurons, at least at the concentrations analyzed. However, microglia could be stimulated by MCP-1 to generate certain factors which we did not analyze or which we simply may not know about. In order to further explore this possibility, microglia were treated with MCP-1 while co-cultured with primary neurons, and no neuronal damage was observed under these conditions, further suggesting a lack of a change in microglia that can be toxic to neurons.

The results presented here indicate that the changes caused by MCP-1 in microglia may not be responsible for the neuronal damage observed in certain situations where MCP-1 expression is elevated, and support the hypothesis which proposes a neuroprotective role for MCP-1.

## Methods

### Reagents

Fetal calf serum (< 10 EU endotoxin per mL), basal medium Eagle, neurobasal medium (NBM), DMEM, DMEM-F12 and the B27 without antioxidants supplements for cell cultures were from GIBCO Life Technologies (Carlsbad, CA, USA). LPS from Escherichia coli 0111:B4 for cell treatments and glutamine, gentamicin, penicillin and streptomycin for cell cultures were from Sigma (St. Louis, MO, USA). Recombinant rat MCP-1 for cell treatments was from Peprotech (Rocky Hill, NJ, USA). Trizol^© ^for RNA isolation, Taq polymerase for cDNA synthesis and cDNA synthesis reagents were from Invitrogen (Carlsbad, CA, USA).

### Microglial cultures

All experimental protocols followed the guidelines of the Animal Welfare Committee of the Universidad Complutense according to European legislation (2003/65/EC).

Rat cortical microglial cells were obtained as described previously [18]. Briefly, 1-day-old Wistar rats (Harlan Iberica) were used. Enriched cultures of microglia were prepared from primary mixed cultures of rat cortical glial cells, plated in T-75-cm^2 ^flasks in DMEM containing 10% FCS and antibiotics (100 IU/ml penicillin and 100 mg/ml streptomycin; Sigma), and incubated at 37°C in a humidified atmosphere containing 5% CO_2_. Briefly, microglial cells were detached from the astrocyte monolayer by gentle shaking 11-13 days after the dissection. The cells were plated at 4·10^5 ^cells/ml in 6.5 mm Transwell^© ^inserts (100 μl/well), 24 (500 μl/well) or 96 (100 μl/well)-well plates. Under these conditions, the cultures were 95-98% Mac-1 positive. All experiments were carried out in 10% FCS/DMEM-F12.

### Neuronal cultures

Primary cultures of cortical neurons were prepared as described [19], with some modifications. Brains were removed from fetal Wistar rats (Harlan Iberica) at embryonic day 16, and the cortical area was dissected. Neurons were mechanically dissociated in 80% basal medium Eagle containing 33 mmol/L glucose, 2 mmol/L glutamine, 16 mg/L gentamicin, 10% horse serum, and 10% fetal calf serum and plated at 1·10^6 ^cells/ml in poly-L-lysine-precoated, 12, 24 or 96-multiwell plates. The medium was replaced 24 h later with serum-free NBM supplemented with 0.5 mmol/L glutamine and complete B27 without antioxidants to reduce glial contamination and after 4 days 50% of the medium was replaced with fresh NBM. Cultures consisted of 98 ± 2% NeuN-positive cells. After 9 days in vitro, inserts (0.4 μm pore size) containing microglia in 10% FCS/DMEM-F12 were placed over the neurons (this system allowed for the transfer of material from one side of the membrane to the other while preventing direct contact of both cell cultures). 24 h later, the media in the inserts were replaced by new ones containing different treatments. After 24 h cell viability was assessed by LDH measurements.

### Nitrites Measurement

NO production was measured indirectly by nitrite measurement in the cell culture media. An aliquot of the cell culture media (80 μl) was mixed with 40 μl of Griess reagent and the absorbance was measured at 550 nm.

### Cell viability

Cell viability was assessed by measurement of released lactate dehydrogenase (LDH), using the CytoTox-96 kit from Promega (Madison, WI, USA) according to the manufacturer's instructions.

### IL-1β Measurement

IL-1β levels in the incubation medium were detected using a specific enzyme-linked immunosorbent assay (ELISA) for rat IL-1β, carried out according to the manufacturer's instructions (R&D Systems, Inc.). Briefly, the medium was collected from the microglial cells and stored at -80°C until the day of the assay (avoiding repeated freeze-thaw cycles). A standard curve was generated during each assay in the concentration range 0-1,000 pg/ml using the rat IL-1β standard provided in the kit. The minimum detectable dose of IL-1β was 5 pg/ml.

### IL-6 Measurement

IL-6 levels in the incubation medium were detected using a specific enzyme-linked immunosorbent assay (ELISA) for rat IL-6, carried out according to the manufacturer's instructions (BD Biosciences). Briefly, the medium was collected from the microglial cells and stored at -80°C until the day of the assay (avoiding repeated freeze-thaw cycles). A standard curve was generated during each assay in the concentration range 0-5,000 pg/ml using the rat IL-6 standard provided in the kit. The assay detection limits were of 78-5000 pg/mL.

### BrdU Incorporation Assay

DNA synthesis was measured using a bromodeoxyuridine (BrdU) Cell Proliferation Kit (Calbiochem, Darmstadt, Germany). BrdU labeling solution was added to the cells in combination with the different treatments and incubated for 24 h. After removal of the culture medium, the cells were fixed, permeabilized and the DNA denatured. Anti-BrdU antibody was then added before the addition of the mouse IgG-peroxidase conjugate. The signal was developed with tetramethylbenzidine solution in darkness. Absorbance in each well was measured using a spectrophotometric plate reader at 450 nm with a reference wavelength at 595 nm.

### mRNA analysis

Total cytoplasmic RNA was prepared from cells using TRIZOL^© ^reagent (Invitrogen); aliquots were converted to cDNA using random hexamer primers. Quantitative changes in mRNA levels were estimated by real time PCR(Q-PCR) using the following cycling conditions: 35 cycles of denaturation at 95°C for 10 s, annealing at 58-61°C for 15 s depending on the specific set of primers, and extension at 72°C for 20 s. Reactions were carried out in the presence of SYBR green (1:10000 dilution of stock solution from Molecular Probes, Eugene, OR, USA), carried out in a 20- μL reaction in a Corbett Rotor-Gene (Corbett Research, Mortlake, NSW, Australia). The primers used for NOS2 were: forward: 5'-AGCA ACA TTT GGC AAT GGAGAC TGC-3' and reverse: 5'-AGC AAA GGC ACA GAA CTG AGG GTA-3'. The primers used for MIP1α were: forward: 5'-CAG AAC ATT CCT GCC ACC TGC AAA-3' and reverse: 5'-AGG AAT GTG CCC TGA GGT CTT TCA-3'. The primers used for Cyclin D1 were: forward: 5'-TGC TGC AAA TGG AAC TGC TTC TGG-3' and reverse: 5'-AAG GTC TGT GCA TGT TTG CGG ATG-3'. The primers used for CDK4 were: forward: 5'-ACG CCT GTG GAT ATG TGG AGT GTT-3' and reverse: 5'-AGT CGT CTT CTG GAG GCA ATC CAA-3'. The primers used for PCNA were: forward: 5'-AGC AAC TTG GAA TCC CAG AAC AGG-3' and reverse: 5'-TAA GGT CCC GGC ATA TAC GTG CAA-3'. The primers used for IL1β were: forward: 5'-ACC TGC TAG TGT GTG ATG TTC CCA-3' and reverse: 5'-AGG TGG AGA GCT TTC AGC TCA CAT-3'. The primers used for IGF-1 were: forward: 5'-CCG CTG AAG CCT ACA AAG TC-3' and reverse: 5'-GGA AAT GCC CAT CTC TGA AA-3'. The primers used for bFGF were: forward: 5'-GAA CCG GTA CCT GGC TAT GA-3' and reverse: 5'-CCG TTT TGG ATC CGA GTT TA-3'. The primers used for IL6 were: forward: 5'-AAC TCC ATC TGC CCT TCA GGA ACA-3' and reverse: 5'-AAG GCA GTG GCT GTC AAC AAC ATC-3'. The primers used for GAPDH were: forward: 5'-TGC ACC ACC AAC TGC TTA GC-3 and reverse: 5'-GGC ATG GAC TGT GGT CAT GAG-3'. Relative mRNA concentrations were calculated from the take-off point of reactions using included software, and GAPDH levels were used to normalize data.

### Cell morphology

Microglia morphology was analyzed by phase contrast microscopy. Images were obtained on a Nikon Eclipse Ti-S (Tokyo, Japan) microscope equipped with a Digital Sight digital camera and NIS-Elements imaging software.

### Statistical analysis

All experiments were done at least in triplicate. When more than two experimental groups were present in the same experiment, data were analyzed by one-way ANOVA followed by Newman-Keuls multiple comparison tests and p values < 0.05 were considered significant. When two experimental groups were present in the same experiment, data were analyzed by unpaired t tests, and p values < 0.05 were considered significant.

## Results

### MCP-1 induces microglia proliferation

**Figure 1:**
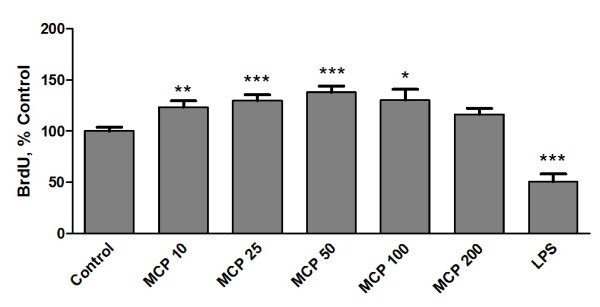
**MCP-1 induces microglia proliferation**. Microglia were incubated for 24 h with fresh media (control), MCP-1 (10-200 ng/ml) or LPS 0.1 μg/ml. Cell proliferation was assayed using a BrdU kit as described in the Methods section. Data are expressed as percentage of control values (set to 100%); ***p < 0.0001 versus control, **p < 0.001 versus control, *p < 0.005 versus control. Data are means ± SE of n = 12 replicates per group.

A BrdU immunoassay was used to evaluate the proliferation of microglia in response to MCP-1. Different concentrations of MCP-1 (10-200 ng/ml) or LPS (0.1 μg/ml) were added to the culture medium and the cells were incubated for 24 h in the presence of BrdU. After this treatment, we could detect a higher degree of BrdU incorporation in the microglia treated with MCP-1. The changes were concentration-dependent up to the concentration of 50 ng/ml. On the other hand, LPS treatment reduced microglia proliferation. (Figure 1)

### Quantification of cell cycle-dependent transcripts

**Figure 2:**
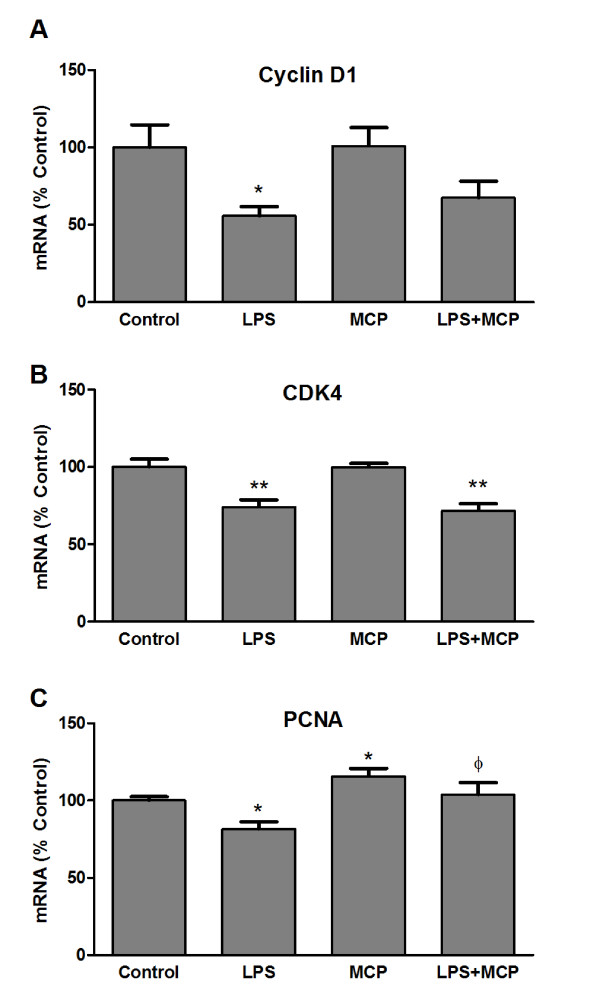
**Expression of cell cycle proteins**. Microglia were incubated for 24 h with fresh media (control), LPS 0.1 μg/ml, MCP-1 50 ng/ml, or a combination of LPS and MCP-1 at the indicated concentrations. RNA was isolated and cyclin D1 (A), CDK4 (B) and PCNA (C) mRNA levels determined by RT-PCR. Data are expressed as percentage of control values (set to 100%); **p < 0.001 versus control, *p < 0.005 versus control, p < 0.005 versus LPS. Data are means ± SE of n = 6 replicates per group.

After observing MCP-1 alteration of microglia proliferation it became interesting to study if the expression of different proteins involved in the regulation of the cell cycle was also modulated by MCP-1. In accordingly with what we observed in BrdU studies, LPS treatment caused a reduction in mRNA levels of cyclin D1, cyclin-dependent kinase 4 (CDK4) and proliferating cell nuclear antigen (PCNA). On the other hand, MCP-1 elevated PCNA mRNA indicating that this treatment induces microglia proliferation. However, no modifications could be detected by MCP-1 treatment of cyclin D1 or CDK4 (Figure 2).

### Microglia activation

**Figure 3:**
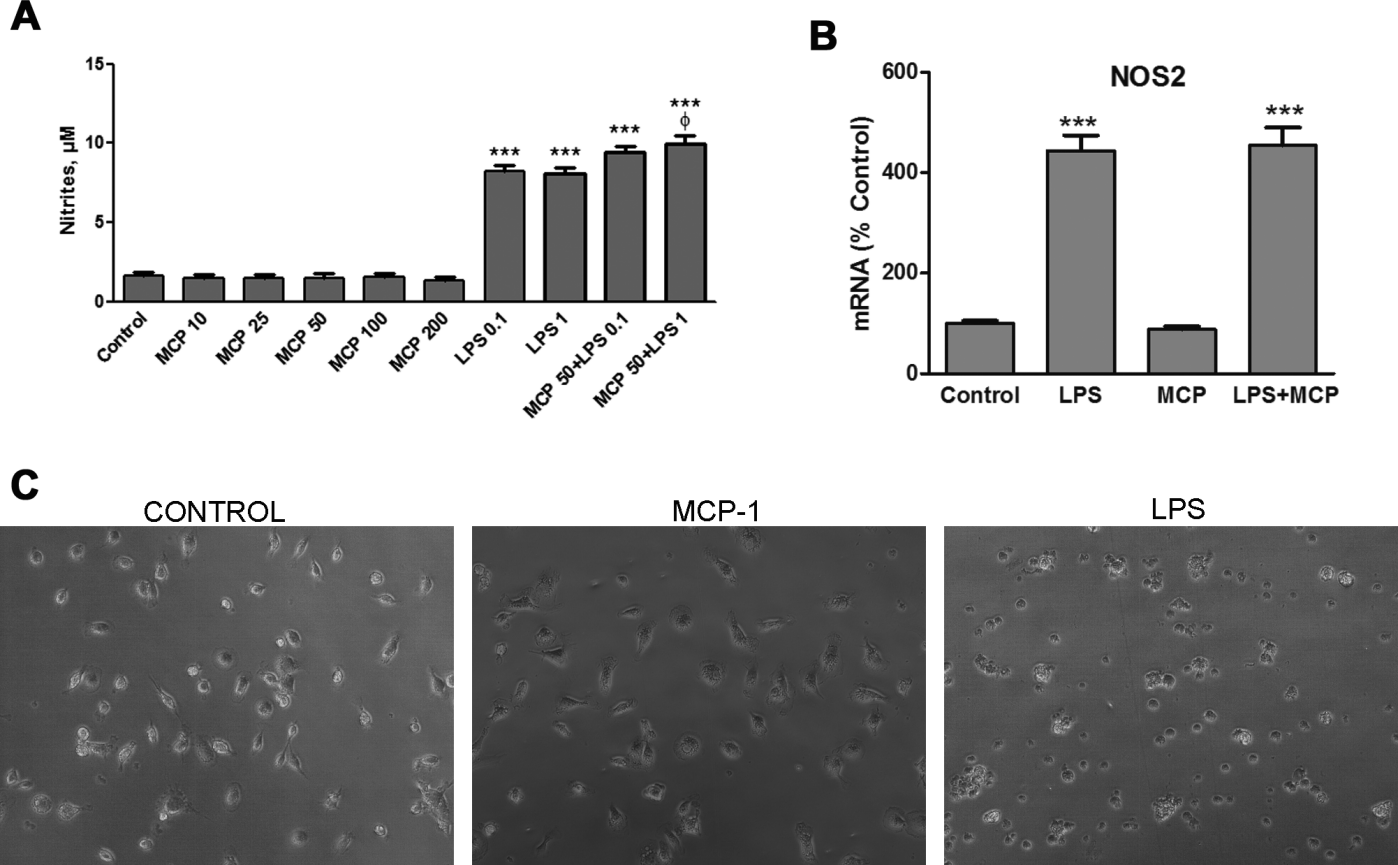
**Microglia activation**. (A) Nitrite levels were measured in microglial media after 24 h incubation with fresh media (control), MCP-1 (10-200 ng/ml), LPS (0.1, 1 μg/ml) or MCP-1 50 ng/ml in combination with LPS. Data are expressed as μM of nitrites and are expressed as mean ± SE for n = 10 replicates per group. ***p < 0.0001 versus control, p < 0.005 versus LPS 1 μg/ml (a). (B) Microglia were incubated for 24 h with fresh media (control), LPS 1 μg/ml, MCP-1 50 ng/ml or the combination of LPS and MCP-1 at the indicated concentrations. RNA was isolated and NOS2 mRNA levels determined by RT-PCR. Data are expressed as percentage of control values (set to 100%); ***p < 0.0001 versus control. Data are expressed as mean ± SE for n = 6 replicates per group. (C) Representative images corresponding to microglia incubated for 24 h with fresh media (control), MCP-1 (50 ng/ml) or LPS 1 μg/ml. The images shown are representative of experiments done on three separate microglial preparations.

The induction of the enzyme NOS2 is considered to be a marker of those microglial modifications that lead to an inflammatory response. To study this possibility, we treated microglia cultures with MCP-1 and measured the concentration of nitrites present in the culture media, as an indirect way to detect modifications of NOS2 activity and the subsequent release of nitric oxide. Under these conditions we could not detect modifications of nitrite concentration by MCP-1 treatment (Figure 3A).

It was also interesting for us to analyze the possible exacerbating effect of MCP-1 in the presence of a different microglia-activating agent. An approach to this was made by co-incubating the cells with different concentrations of LPS. We observed a small but significant increase when MCP-1 was added to the higher concentration of LPS used (Figure 3A); however we could not detect similar differences in the other experiments described below.

As a confirmation of the nitrite differences, mRNA levels of NOS2 in microglia were measured by RT-PCR. For this we chose the concentration of MCP-1 with the highest proliferative effect. Under the conditions used (50 ng/ml, 24 h), MCP-1 did not modify the expression of NOS2, nor did it alter the induction caused by LPS treatment (Figure 3B).

Morphological alterations of microglia are also a good way to evaluate the effect of different agents on this type of cell. Enlargement of the microglial cell body and loss of ramifications, developing an amoeboid shape, are commonly caused by LPS or other toxins [20]. While those changes could be easily detected in LPS-treated cells, no modifications in the appearance of MCP-1-treated cells could be appreciated (Figure 3C).

### Expression of inflammatory cytokines

**Figure 4:**
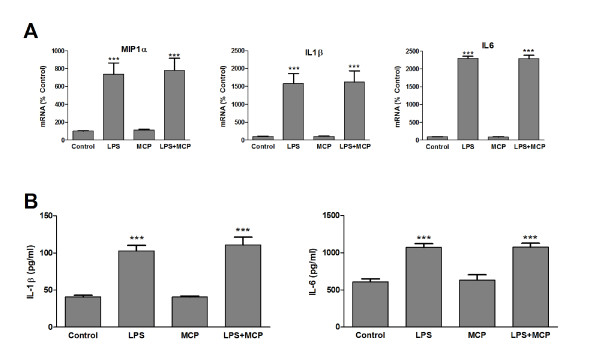
**Expression of pro-inflammatory cytokines**. (A) Microglia were incubated for 24 h with fresh media (control), LPS 1 μg/ml, MCP-1 50 ng/ml or a combination of LPS and MCP-1 at the indicated concentrations. RNA was isolated and MIP-1α, IL-1β and IL6 mRNA levels determined by RT-PCR. Data are expressed as percentage of control values (set to 100%); ***p < 0.0001 versus control. Data are expressed as mean ± SE for n = 6 replicates per group. (B) Microglia were incubated for 24 h with fresh media (control), LPS 1 μg/ml, MCP-1 50 ng/ml or a combination of LPS and MCP-1 at the indicated concentrations. IL-1β and IL-6 levels in the media were assessed by ELISA. ***p < 0.0001 versus control. Data are expressed as mean ± SE for n = 10 replicates per group.

Our main goal was to analyze if MCP-1 can provoke some change in microglia that results in toxicity to neurons. For this purpose we measured the expression and synthesis of some pro-inflammatory cytokines such as interleukin 1β (IL-1β) or interleukin 6 (IL-6) as well as the chemokine macrophage inflammatory protein 1 alpha (MIP-1α). MCP-1 alone or in combination with LPS was added to microglia cultures and the cells were incubated for 24 h. After this, mRNA was isolated and analyzed by RT-PCR. MCP-1 caused no significant changes in any of the cytokines studied, nor did it alter the elevations caused by LPS (Figure 4A). Release of IL-1β and IL-6 into the culture media was also measured by ELISA and no modification could be detected as a result of MCP-1 treatment (Figure 4B).

### Neuronal toxicity

**Figure 5:**
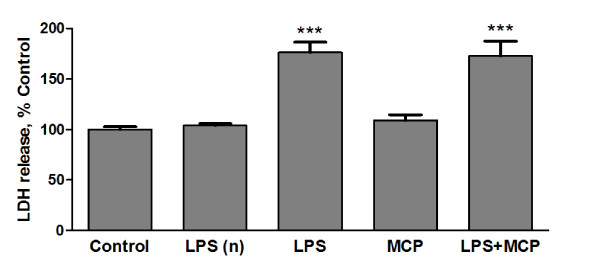
**Neuronal toxicity**. Microglia plated on transwell membranes were transferred to wells containing primary neurons. After 24 h, the microglia were treated for a further 24 h with fresh media (none), LPS 1 μg/ml, MCP-1 50 ng/ml or a combination of LPS and MCP-1 at the indicated concentrations. After treatment, inserts were removed and cell viability was assessed by measurement of LDH in the neuronal media. Inserts without microglia were treated with LPS (LPSn) under the same conditions as those described above for microglia. Data are expressed as percentage of control values (set to 100%); ***p < 0.0001 versus control. Data are expressed as mean ± SE for n = 12 replicates per group.

Since we could not observe modification by MCP-1 of any of the characteristic activation markers evaluated, we decided to analyze the direct effect of MCP-1-treated microglia on neurons. Primary cortical neurons were cultured for 9 days. At this point, microglia inserts were placed over the neurons. As previously described, MCP-1 was added to microglial cultures alone or in combination with LPS, and 24 h later LDH levels were measured in the neuronal culture media. While the addition of LPS to microglial cultures caused neuronal death, MCP-1 did not affect the viability of the neurons nor did it alter LPS effects (Figure 5).

The direct effect of LPS on neurons was evaluated by treating inserts without microglia in the same conditions as those containing microglia. This procedure did not result in significant alterations of neuronal LDH.

### Production of trophic factors

**Figure 6:**
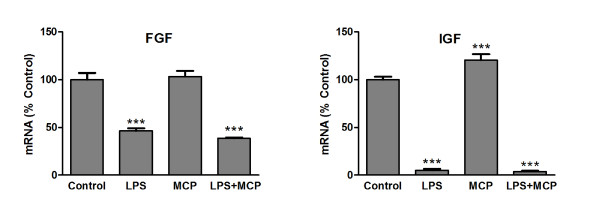
**Production of trophic factors**. Microglia were incubated for 24 h with fresh media (control), LPS 1 μg/ml, MCP-1 50 ng/ml or a combination of LPS and MCP-1 at the indicated concentrations. RNA was isolated and bFGF (A) and IGF-I (B) mRNA levels determined by RT-PCR. Data are expressed as percentage of control values (set to 100%); ***p < 0.0001 versus control. Data are expressed as mean ± SE for n = 6 replicates per group.

Since MCP-1 does not appear to induce production of potentially neurotoxic agents such as nitric oxide or proinflammatory cytokines, it could cause harmful effects by reducing the production of trophic factors. mRNA levels for basic fibroblast growth factor (FGF) and insulin-like growth factor 1 (IGF) were measured by RT-PCR in microglia treated with MCP-1 alone or in combination with LPS for 24 h. Under these conditions, LPS treatment caused a great reduction in the production of both proteins' mRNAs while MCP-1 did not cause any modification for FGF (Figure 6A) and caused an increase for IGF (Figure 6B).

## Discussion

The data presented here analyze microglial activation as a consequence of MCP-1 treatment. While no morphological changes or production of inflammatory mediators could be detected, we found that MCP-1 stimulates the formation of new microglia. This suggests that MCP-1 could elevate microglia concentrations by attracting these cells and also by facilitating their proliferation, but it would not be associated with their activation or with the generation of neurotoxic agents.

Since MCP-1 activity can be modified by plasmin or matrix metalloproteinases [21,22], we cannot discard the possibility that such modification is a requirement for MCP-1 to exert the effects observed.

After its discovery as a chemokine, and particularly over the last few years, MCP-1 has proven to be a key mediator in different processes. Some of them are related to its ability to attract cells, such as directing the migration of neural progenitors [23], while others seem to be independent of its chemoattractant abilities and suggest that MCP-1 may also participate as a modulator of neuronal communication [14,24], neuronal regeneration [15], angiogenesis, hematopoiesis [25] or immunoregulation [16].

Besides these different roles of MCP-1, there are several studies focused on its involvement in neuro-inflammatory reactions [26]. Since MCP-1 attracts those cells necessary for the development of an inflammatory response, the blockade of this attraction would reduce inflammation and all the changes associated to it. However, inhibition of MCP-1 production or of its activity should be carefully considered as a therapeutic target, mainly because some of its actions could be necessary for homeostasis maintenance or simply remain yet unknown. Based on this, we sought here to elucidate, at least in part, if MCP-1 can directly cause activation of the main immune cells in brain, and if this could contribute to the death of neurons.

While MCP-1's ability to attract leukocytes [27] and other types of cells such as microglia [12] is well known, its proliferative effect on microglia has not been so intensely investigated. Studies performed on human astrocyte cultures have also shown the ability of MCP-1 to induce proliferation of these cells [28], but to our knowledge this is the first study to describe the induction of microglia proliferation by MCP-1.

We tested the effects of different concentrations of MCP-1 on nitrite production and no modification was observed for any concentration. A concentration of 50 ng/ml was the lowest one of those tested to induce the maximal degree of proliferation detected. Based on this we chose that concentration for further experiments. At similar concentrations we had previously observed MCP-1 to cause protective changes in neuronal cultures [29].

The morphology of the cells, together with the measurements of MIP-1α, IL-1β, IL-6 and NOS2 expressions and synthesis indicates that MCP-1 under the conditions used does not induce pro-inflammatory changes in microglia.

Other authors have reported that MCP-1 induces production of IL-1β and TNFα in mice microglial cultures as well as death of neurons exposed to MCP-1-activated microglia [30]. However, we could not observe similar effects in our cultures. This may be due to inter-species differences or to some other unidentified factors we are not yet aware of. Further experimentation may help us to identify the source of this dissimilarity.

Also, certain in vivo studies have shown that MCP-1 activates spinal microglial cells [31] and that MCP-1 blockade may reduce microglial activation [32]. But due to the nature of the experimental models used in these studies, where microglia are not isolated, we cannot conclude that MCP-1 by itself causes activation of microglia. The use of cultures, despite all its disadvantages (i.e. isolation of cells from their natural environment) provides a better way to analyze the direct effect of any agent on a particular type of cell. This was precisely our goal: to study the direct effect of MCP-1 on microglia in the absence of other cells that might also affect microglial behaviour.

The situation could be explained in a simplistic way by considering that MCP-1 may attract microglia (and maybe induce their proliferation), but other factors could be responsible for their activation. Furthermore, the attraction of microglia could help healing injuries through transformation of these cells into other cell types [33].

It was of particular interest for us to find that LPS had an effect opposite to that of MCP-1, reducing the proliferation of microglia. Other groups have described no proliferative effect of LPS or a reduction of proliferation when combined with IFNγ [34,35]. These changes have been classified by some authors as a resemblance of a chronic activation of microglia [36] which leads to the degeneration of these cells and a subsequent loss of the support they provide for neurons that will also eventually result in neuronal death.

This is in agreement with the reduction in expression of the trophic factors FGF and IGF that we could detect as a result of LPS treatment of microglia. Microglia have previously been shown to be a source of FGF [37] and IGF [38]. FGF stimulates neurite outgrowth of different types of neurons and supports their survival [39,40] while IGF also is known to protect neurons and to promote proliferation of neuronal progenitors [41] as well as glial cells [42,43]. Interestingly, we detected an increase of IGF mRNA after treating microglia with MCP-1. IGF has also been proposed as a mediator of the protection MCP-1 provides for retinal ganglion cells in an experimental glaucoma model [44]. In this study [44], the authors showed that while elevated concentrations of MCP-1 can be neurotoxic, lower ones have the opposite effect. They also indicated how, in the absence of glial cells, neuroprotective actions of MCP-1 are not observed, suggesting that these cells may help MCP-1 to protect neurons. In according with this idea, the proliferation of microglia that we describe here would facilitate MCP-1's neuroprotective potential. This offers the possibility to explore this event in vivo, and to analyze if there are any differences between resident and bone marrow-derived microglia, since MCP-1 acts on both of these types of cells [45].

Altogether, we can hypothesise that MCP-1, like many other regulators of brain cell function, may exert a maintenance role in the CNS at constitutive concentrations; however, as a result of certain disturbances, its production can be altered leading to different changes, some of which could have deleterious consequences.

As mentioned above, our co-culture model allowed us to test the possible consequences of microglial exposure to MCP-1 on neurons. This study confirmed the information obtained from the cytokine measurements, suggesting that MCP-1 does not appear to cause microglial modifications that can damage neurons.

Our previous studies have demonstrated induction of MCP-1 in astrocytes by the neurotransmitter noradrenaline (NA) [29]. Our interest on NA is based on its anti-inflammatory effects, which have been confirmed by both *in vivo *[46,47] and *in vitro *[19,48] studies. These effects help protect neurons against different injuries, and reduction of NA levels in brain seems to facilitate the progression of certain neurodegenerative diseases such as Alzheimer's [49] and Parkinson's diseases [50]. Recently, a role for NA in the progression of multiple sclerosis has also been described [51].

After finding that NA causes a large production of MCP-1 in astrocytes, it seemed reasonable to consider MCP-1 as a mediator of some noradrenergic effects. In fact we could demonstrate that noradrenaline induction of MCP-1 in astrocytes can protect neurons against excitotoxic damage [29]. Other authors have also described MCP-1 as a neuroprotective agent against excitotoxicity [11,13] or in neurodegenerative diseases such as Alzheimer's disease [12].

## Conclusions

In summary, the goal of this study was to analyze the possible involvement of MCP-1 in the neuronal damage observed in certain situations where an inflammatory response takes place, and particularly, if this is mediated by the activation of microglia. Our results indicate that direct incubation of MCP-1 with cultured microglia stimulates microglial proliferation but it does not appear to cause modifications that interfere with neuronal viability. According to this, inhibition of MCP-1 as a way to protect neurons in certain pathologies should be carefully considered. This chemokine may not be directly responsible for the neuronal damage characteristic of those situations, and control of its function may interfere with other pathways it regulates.

## Competing interests

The authors declare that they have no competing interests.

## Authors' contributions

AEH carried out the cell assays, RT-PCR, data acquisition and helped to draft the manuscript. BG and JCL contributed to analysis and interpretation of data, drafting the manuscript and revising it critically. JLMM performed cell cultures and treatments, conceived the study and participated in its design and coordination and helped to draft the manuscript. All authors read and approved the final manuscript.
